# A Facile and Efficient Synthesis of Diaryl Amines or Ethers under Microwave Irradiation at Presence of KF/Al_2_O_3_ without Solvent and Their Anti-Fungal Biological Activities against Six Phytopathogens

**DOI:** 10.3390/ijms140918850

**Published:** 2013-09-12

**Authors:** Liang-Zhu Huang, Pan Han, You-Qiang Li, Ying-Meng Xu, Tao Zhang, Zhen-Ting Du

**Affiliations:** 1 College of Science, Northwest A & F University, Yangling 712100, China; E-Mails: hlz15106006@163.com (L.-Z.H.); hanpan_093@163.com (P.H.); lyq812322926@163.com (Y.-Q.L.); dirk41414141@126.com (Y.-M.X.); fuzitong@163.com (T.Z.); 2 Key Laboratory of Synthetic Chemistry of Natural Substances, Shanghai Institute of Organic Chemistry, Chinese Academy of Sciences, Shanghai 20032, China

**Keywords:** microwave-assisted organic synthesis, diaryl amine, diaryl ether, KF/Al_2_O_3_

## Abstract

A series of diaryl amines, ethers and thioethers were synthesized under microwave irradiation efficiently at presence of KF/Al_2_O_3_ in 83%–96% yields without any solvent. The salient characters of this method lie in short reaction time, high yields, general applicability to substrates and simple workup procedure. At the same time, their antifungal biological activities against six phytopathogen were evaluated. Most of the compounds (**3b**, **3c**, **3g**–**o**) are more potent than thiophannate-methyl against to *Magnaporthe oryzae*. This implies that diaryl amine or ether moiety may be helpful in finding a fungicide against *Magnaporthe oryzae*.

## 1. Introduction

Microwave-assisted organic synthesis (MAOS) has been one of the most exciting areas of interest on which many reviews have been published in last three decades [1–4]. Numerous reactions, including condensations [5–8], cycloadditions [9–12], heterocycles formations [13–15], and metal catalyzed cross-coupling [16,17] have been explored under microwave conditions. Some of these have been applied to medicinal chemistry and total syntheses of natural products [18–20]. MAOS can facilitate the discovery of new reactions and reduce cycle time in optimization of reactions. In addition, it serves to expand chemical space in compound library synthesis.

**Figure 1:**
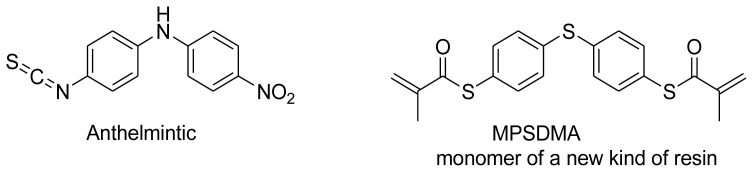
Representive diaryl heteroatom molecules.

Diaryl heteratom moities can be found from natural products, pharmaceuticals or optical materials [21,22] (Figure 1). Traditionally, they are prepared through a copper-assisted Ullmann reaction by intermolecular SNAr way. However, the key concerns of this chemical operation are harsh conditions (reaction temperature >200 °C) and troublesome residue stemming from a stoichiometric amount of copper [23] in terms of chemical waste. Palladium and copper complexes with various kinds of ligands have been studied fully for the cross-coupling between heteroatom (N, O, S) with aryl halide [24–27]. Transition metal catalysis (including Cu [28], Ni [29,30], Fe [31–33]) are involved as a complementary means of cross-coupling. However, the researchers still are confronted with the cost of precious metal and metal residue in products. In our pursuing new heterocyclic structures which serve as potential bioactive compounds in agriculture, we discovered a new palladium catalyzed cyclization of diazonium salts to form dibenzo[*d*]furan [34] and 6*H*-benzo[*c*]chromenes [35]. In preparing the substrates of such kinds of reaction patterns, we need to rapidly obtain a quantity of the derivatives of diaryl amine, ether and thioether. The existing methods in the literature seem tedious, laborious or not applicable. Therefore, there is still a need for innovation in such a general chemical transformation in order to provide corresponding structures effectively and on a feasible scale. Herein, we wish to report an improved method in preparation of these kinds of substrates under microwave irradiation.

## 2. Results and Discussion

Initially, the *o*-nitro chlorobezene and aniline were chosen as starting materials of model reaction. Thus, the different bases and solvents were also involved in this test and the results are summarized in Table 1. The reaction was performed in polar non-protonic solvent and at presence of K_2_CO_3_ as base in refluxing temperature. To our regret, the conversion rate of both were below 45%, even after 12 h. Following this, we introduced microwave irradiation to the system: the conversion rate increased considerably. Then, several bases such as (K_2_CO_3_Table 1, entry 3, NaOH, entry 5, KF/Al_2_O_3_ entry 8 and without base entries 6 and 7) were screened under microwave irradiation. Na_2_CO_3_ did not show a positive effect on this conversion and NaOH showed a worse result. We suspected that the complication of the products was due to the high concentration of NaOH which will attack chloride directly. The solvent-free system was also performed and the yield is higher than in DMF because of the latter’s higher reaction temperature. Finally, a composite solid base KF/Al_2_O_3_ was chosen as the best catalyst for this reaction. A literature survey revealed that KF/Al_2_O_3_ showed wide spectrum applications in base catalyzed reactions [36–38].

Under these optimized reaction conditions, we next examined the scope of KF/Al_2_O_3_ catalyzed coupling of *o*-nitrophenylchloride **1** and a wide spectrum of substrates such as amines, phenols and thiophenols **2** for the synthesis of substituted analogues of diphenyl amine. The results are summarized in Table 2. A wide range of structurally diverse amines, phenols, and thiophenols (Table 2) can be coupled with *o-*nitrohalobenzene under this protocol to give the corresponding substituted diaryl hetero ethers in excellent yields. It should be noted that the reactants need preheat to melt before microwave irradiation. Among them, bromo (Table 2, entries 4 and 9) and chloro (Table 2, entries 5 and 14) groups can be tolerated. The bromo and chloro moieties could be functionalized to boric acid or stannane easily, so our method effectively allows the preparation of halo diaryl hetero ethers. Thus, all the products in our reactions listed in Table 2 were easily characterized on the basis of physical and spectral data and also by comparison with authentic samples. All products (Table 2) were fully characterized by spectroscopic methods, as well as by the comparison of the spectral data with reported values.

Having obtained these 15 compounds, their antifungal activities (**3a**–**o**) against six phytopathogenic fungi (*i.e.*, Cytospora mandshurica, Curvularia lunata, Magnaporthe oryzae, Gloeosporium fructigenum, Alternaria alternate, Fusarium graminearum) were investigated at the concentration of 100 μg/mL *in vitro* by poisoned food technique [39]. Thiophanate-methyl, which is structurally similar to these compounds and a commercially available agricultural fungicide, was used as a positive control at 100 μg/mL. For each treatment, three replicates were conducted. The radial growths of the fungal colonies were measured and the data were statistically analyzed. The inhibitory effects of the test compounds on these fungi *in vitro* were calculated by the formula:

(1) Inhibition rate (%)=(C-T)×100/C

where *C* represents the diameter of fungi growth on untreated Potato Dextrose Agar (PDA), and *T* represents the diameter of fungi on treated PDA.

As outlined in Table 3, all the analogues of diaryl amine (entries **3a**–**g**) showed only fairly good antifungal activities comparing with thiophannate-methyl. As for *Alternaria lternata* and *Fusarium graminearum*, compounds (**3a**, **3d**–**f**), they show unsatisfactory activity. As for compounds **3d**–**f**, they were almost inactive to the phytopathogenic fungi. Diaryl ethers (entries **3h**–**k**) also showed only fairly good antifungal activities. It should be noted that the inhibition rate of **3h** to *Curvularia lunata* is as high as 62.67%, compared with the one of thiophannate-methyl, 37.95%. As for diaryl thioethers (**3l**–**o**), they showed moderate antifungi bioactivities. On the other hand, most of the compounds (entries **3b**, **3c**, **3g**–**o**) are more potent than thiophannate-methyl against *Magnaporthe oryzae*. This implies that diaryl moiety may be more helpful in fungicide against *Magnaporthe oryzae*.

## 3. Experimental Section

### 3.1. Typical Synthetic Procedure

A well dispensed mixture of 2-nitrochloro benzene (10 mmol), aniline (10 mmol) and KF/Al_2_O_3_ (2 g) was vigorously stirred and irradiated in microwave reactor (Sineo MAS-II, Shanghai, China) at internal temperature 150 °C for 15 min. Then the reaction mixture was diluted by dichloro methane (60 mL) and the organic layer was washed by saturated aqueous NaHCO_3_ and brine, and dried with anhydrous MgSO_4_. The solvent was evaporated in vacuum and the residue was purified through column chromatography to give **3** (Table 2). The ^1^H-NMR and ^13^C-NMR data were recorded in deutrated chloroform solution with NMR spectrometers (DRX 500, Bruker, Billerica, Massachusetts) if not noted otherwise. The chemical shifts are measured relative to tetramethylsilane (TMS) (δ = 0) or chloroform (δ = 7.26) and the coupling *J* is expressed in Hertz.

#### 3.1.1. 2-Nitrodiphenylamine (**3a**)

Orange solid, mp 74–76 °C (lit. [40], 76–77 °C). ^1^H-NMR: 9.50 (s, 1H), 8.20 (dd, 1H, *J* = 7.2, 1.4), 7.35–7.45 (m, 3H), 7.20–7.30 (m, 4H), 6.78 (t, 1H, *J* = 6.9); ^13^C-NMR: 143.0, 137.9, 134.8, 132.4, 129.7, 126.8, 125.4, 124.4, 117.5, 116.1.

#### 3.1.2. 4′-Methl-2-nitrodiphenylamine (**3b**)

Orange solid, mp 69–70 °C (lit. [41], 69–70 °C). ^1^H-NMR: 2.38 (s, 3H), 6.73 (t, 1H, *J* = 7.8), 7.13–7.16 (m, 3H), 7.22 (d, 2H, *J* = 8.3), 7.33 (t, 1H, *J* = 6.6), 8.19 (dd, 1H, *J* = 8.6, *J* = 1.4), 9.45 (s, 1H). ^13^C-NMR: 21.0, 116.0, 117.1, 124.8, 126.7, 130.3, 132.8, 135.7, 135.8, 135.9, 143.7.

#### 3.1.3. 4′-Methoxy-2-nitrodiphenylamine (**3c**)

Orange solid, mp 88–89 °C (lit. [40,41], 87–88 °C). ^1^H-NMR: 9.41 (s, 1H), 8.19 (d, 1H, *J* = 8.6), 7.30 (t, 1H, *J* = 7.9), 7.20 (d, 2H, *J* = 8.3), 6.90–7.15 (m, 3H), 6.71 (t, 1H, *J* = 7.7), 3.84 (s, 3H). ^13^C-NMR: 157.7, 144.2, 135.6, 132.5, 131.1, 127.3, 126.5, 116.8, 115.6, 114.7, 55.6.

#### 3.1.4. 4′-Bromo-2-nitrodiphenylamine (**3d**)

Orange solid, mp 170–171 °C (lit. [40,41], 168–169 °C). ^1^H-NMR: 6.81 (t, 1H, *J* = 7.8), 7.15–7.21 (m, 3H), 7.39 (t, 1H, *J* = 7.8), 7.52 (d, 2H, *J* = 8.6), 8.21 (dd, 1H, *J* = 1.4, *J* = 8.6), 9.39 (s, 1H). ^13^C-NMR: 115.9, 115.9, 118.1, 118.4, 125.7, 126.8, 132.8, 135.8, 137.9, 142.4.

#### 3.1.5. 4′-Chloro-2-nitrodiphenylamine (**3e**)

Orange solid, mp 170–171 °C (lit. [41], 168–169 °C). ^1^H-NMR (500 MHz, CDCl_3_): 6.83 (t, 1H, *J* = 8.0), 7.15–7.32 (m, 3H), 7.35–7.45 (m, 3H), 8.24 (dd, 1H, *J* = 8.6, 1.5). ^13^C-NMR: 115.9, 118.0, 121.5, 125.6, 126.9, 129.3, 130.1, 135.7, 142.4, 144.1.

#### 3.1.6. 2,4-Dinitrodiphenylamine (**3f**)

Orange solid, mp 158–159 °C (lit. [42], 156–157 °C). ^1^H-NMR: 7.17 (d, 1H, *J* = 9.6), 7.32 (d, 2H, *J* = 7.7), 7.39 (t, 1H, *J* = 7.4), 7.52 (t, 2H, *J* = 7.7), 8.17 (dd, 1H, *J* = 2.6, *J* = 9.6), 9.17 (d, 1H, *J* = 2.6), 9.99 (s, 1H). ^13^C-NMR: 116.1, 124.1, 125.5, 127.8, 129.9, 130.3, 131.1, 136.7, 137.4, 147.1.

#### 3.1.7. 2′-Methyl-2,4-dinitrodiphenylamine (**3g**)

Orange solid, mp 123–124 °C (lit. [43], 124–126 °C). ^1^H-NMR: 2.27 (s, 3H), 6.83 (d, 1H, *J* = 9.6), 7.28 (d, 1H, *J* = 3.6), 7.34 (dd, 2H, *J* = 3.6, *J* = 5.6), 7.39 (t, 1H, *J* = 4.8), 8.15 (dd, 1H, *J* = 2.6, *J* = 9.5), 9.19 (d, 1H, *J* = 2.6), 9.83 (s, 1H). ^13^C-NMR: 17.9, 115.9, 124.2, 126.8, 127.7, 128.5, 130.0, 130.8, 131.9, 134.9, 135.1, 137.2, 147.5.

#### 3.1.8. 2-Nitrophenyl phenyl ether (**3h**)

Yellowish oil, ^1^H-NMR: δ = 8.29 (dd, 1H, *J* = 8.6, 1.4), 7.85 (dd, 1H, *J* = 8.3, 2.3), 7.35–7.45 (m, 3H), 7.20–7.30 (m, 4H). ^13^C-NMR: 157.1, 149.9, 139.5, 134.2, 129.7, 123.5, 122.2, 118.0, 117.3.

#### 3.1.9. 4′-Bromophenyl-2-nitrophenyl ether (**3i**)

Yellow solid, mp 68–69 °C (lit. [44], 71 °C). ^1^H-NMR: 6.92 (dd, 2H, *J* = 2.1, *J* = 6.8), 7.04 (dd, 1H, *J* = 1.0, *J* = 8.4), 7.25 (t, 1H, *J* = 7.6), 7.48 (dd, 2H, *J* = 2.1, *J* = 6.8), 7.54 (t, 1H, *J* = 8.0), 7.96 (dd, 1H, *J* = 1.6, *J* = 8.2). ^13^C-NMR: 117.2, 120.6, 120.9, 123.9, 125.9, 133.1, 134.3, 150.0, 155.2.

#### 3.1.10. 4′-Methylphenyl-2-nitrophenyl ether (**3j**)

Yellow oil, ^1^H-NMR: 7.92–7.96 (m, 1H), 7.45–7.50 (m, 1H), 7.10–7.20 (m, 3H), 6.95–7.00 (m, 3H), 2.37 (s, 3H); ^13^C-NMR: 153.7, 151.7, 141.5, 134.8, 134.4, 131.0, 126.1, 123.0, 120.2, 119.8, 21.2.

#### 3.1.11. 4′-Methoxyphenyl-2-nitrophenyl ether (**3k**)

Yellow solid, mp 47–48 °C (lit., 48 °C). ^1^H-NMR: 3.81 (s, 3H), 6.91 (dd, 3H, *J* = 2.4, *J* = 6.8), 7.02 (dd, 2H, *J* = 2.3, *J* = 6.8), 7.12 (t, 1H, *J* = 7.7), 7.44 (t, 1H, *J* = 7.7), 7.92 (dd, 1H, *J* = 1.6, *J* = 8.2). ^13^C-NMR: 55.7, 115.1, 118.9, 121.2, 122.2, 125.7, 134.0, 140.7, 148.6, 151.9, 156.8.

#### 3.1.12. 2-Nitrodiphenylthioether (**3l**)

Yellow solid, mp 81–82 °C (lit. [45], 80 °C). ^1^H-NMR: 6.86 (dd, 1H, *J* = 1.1, *J* = 8.2), 7.21 (t, 1H, *J* = 7.7), 7.34 (t, 1H, *J* = 7.7), 7.48–7.50 (m, 3H), 7.58 (dd, 2H, *J* = 1.9, *J* = 5.0), 8.22 (dd, 1H, *J* = 1.4, *J* = 8.3). ^13^C-NMR: 125.0, 125.8, 128.3, 130.1, 130.2, 131.0, 133.5, 136.0, 139.5, 144.9.

#### 3.1.13. 4′-Methyl-2-nitrodiphenylthioether (**3m**)

Yellow solid, mp 88–90 °C (lit. [45], 88 °C). ^1^H-NMR: 2.43 (s, 3H), 6.85 (dd, 1H, *J* = 1.0, *J* = 8.2), 7.19 (t, 1H, *J* = 7.7), 7.28–7.35 (m, 3H), 7.46 (d, 2H, *J* = 8.0), 8.22 (dd, 1H, *J* = 1.2, *J* = 9.3). ^13^C-NMR: 21.4, 124.8, 125.8, 127.3, 128.1, 131.0, 133.4, 136.0, 140.1, 140.5, 144.8.

#### 3.1.14. 4′-Chloro-2-nitrodiphenylthioether (**3n**)

Yellow solid, mp 95–96 °C (lit. [45], 94 °C). ^1^H-NMR: 6.86 (dd, 1H, *J* = 1.1, *J* = 8.2), 7.24 (t, 1H, *J* = 7.8), 7.37 (t, 1H, *J* = 7.7), 7.46 (dd, 2H, *J* = 2.2, *J* = 8.8), 7.52 (dd, 2H, *J* = 2.0, *J* = 6.5), 8.23 (dd, 1H, *J* = 1.4, *J* = 8.2). ^13^C-NMR: 125.3, 125.9, 128.2, 129.6, 130.4, 133.6, 136.5, 137.2, 138.8, 145.1.

#### 3.1.15. 4′-Methyl-4-chloro-2-nitrodiphenylthioether (**3o**)

Yellow solid, mp 119–120 °C (lit. [46], 121 °C). ^1^H-NMR: 2.43 (s, 3H), 6.78 (d, 1H, *J* = 8.8), 7.30 (d, 3H, *J* = 7.6), 7.45 (d, 2H, *J* = 8.0), 8.21 (d, 1H, *J* = 2.3). ^13^C-NMR: 21.4, 125.5, 126.7, 129.3, 130.5, 130.6, 130.9, 131.1, 133.5, 135.9, 138.9, 140.8, 144.8.

## 4. Conclusions

In conclusion, a practical KF/Al_2_O_3_ catalyzed synthesis analogue of diaryl heteroatom moties under MWI has been developed. This method offers several advantages, such as high yields, short reaction times, clean reaction profiles, and simple experimental and easy work-up procedures. Fifteen products were tested against six phytopathogenic fungi and their preliminary SAR were analyzed.

## Figures and Tables

**Table 1:** Screen conditions in diaryl amine formation a.

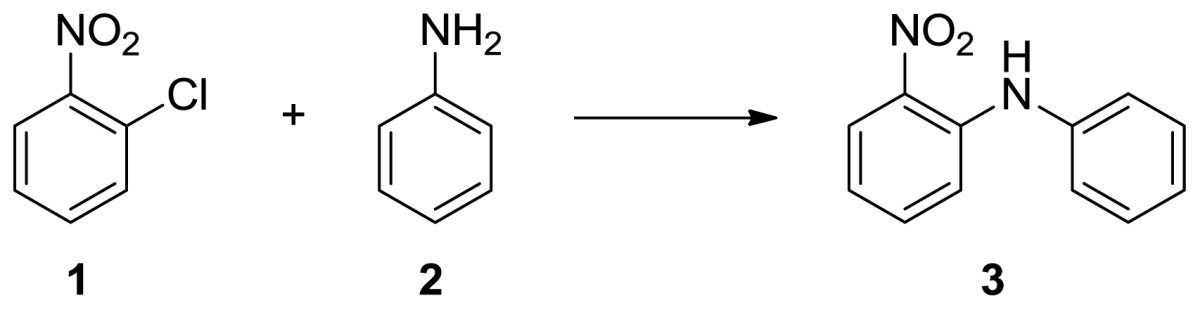				

Entry	Base	Solvent	MWI/Heat	Yield(%) b
1	K_2_CO_3_	DMF	Heat to 80 °C	30
2	K_2_CO_3_	DMA	Heat to reflux	42
3	K_2_CO_3_	DMF	MWI 15 min c	75
4	Na_2_CO_3_	DMF	MWI 15 min c	62
5	NaOH	DMF	MWI 15 min c	47
6	none	DMF	MWI 15 min c	35
7	none	none	MWI 15 min c	56
8	KF/Al_2_O_3_	none	MWI 15 min c	92 b

a The reaction was performed at molar ratio of compound **1** and **2** at 1:1;

b Isolated yields;

c The internal temperature was set as 150 °C on a MAS-II microwave reactor; DMF: *N*,*N*-dimethylformamide; DMA: *N*,*N*-dimethylacetamide; MWI: microwave irradiation.

**Table 2:** Synthesis of diaryl hetero atom moieties under MWI and KF/Al_2_O_3_^a^.

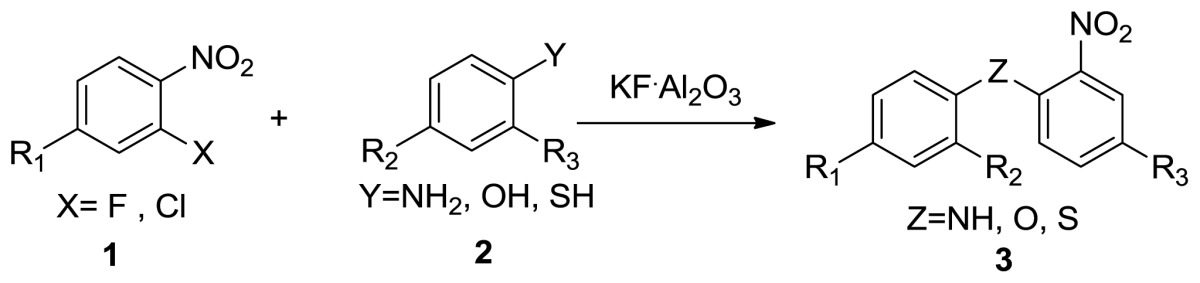					

Entry	R_1_	R_2_	R_3_	Product 3	Yield (%) b
1	H	H	H	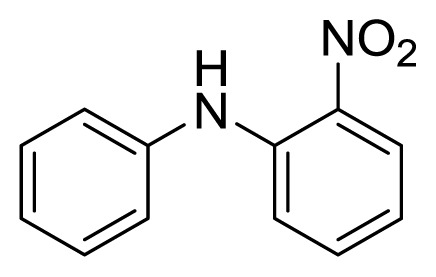 **3a**	92.3 c, 93.5 d
2	H	Me	H	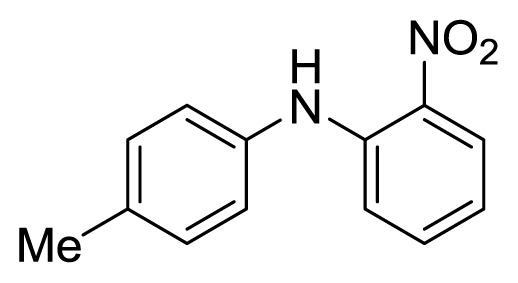 **3b**	94.2 c
3	H	MeO	H	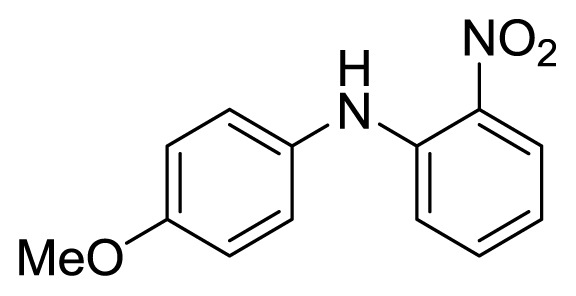 **3c**	100 c,d
4	H	Br	H	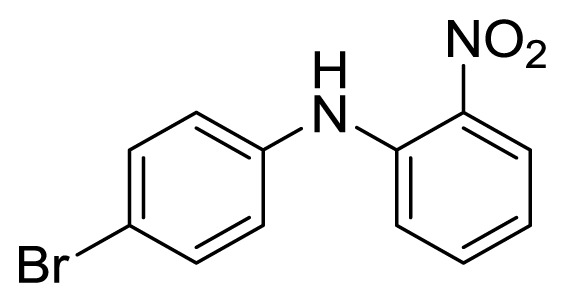 **3d**	85.2 c, 87.0 d
5	H	Cl	H	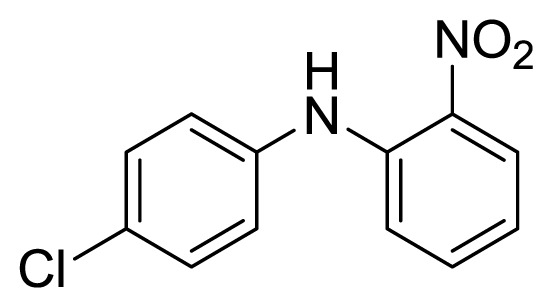 **3e**	83.7 c
6	NO_2_	H	H	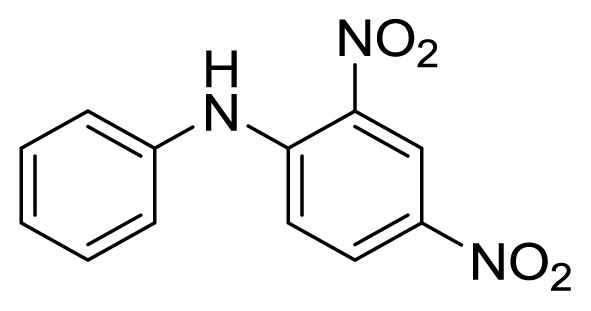 **3f**	93.8 d
7	NO_2_	H	Me	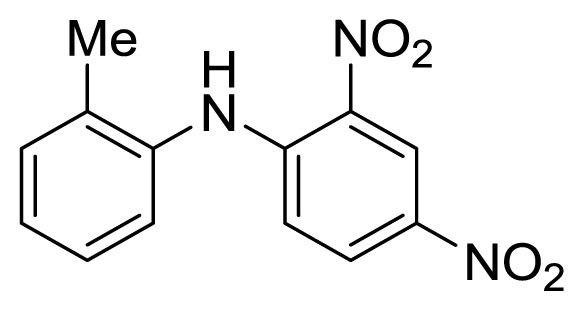 **3g**	95.4 d
8	H	H	H	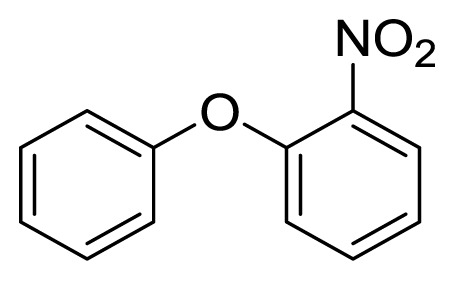 **3h**	91.7 d
9	H	Br	H	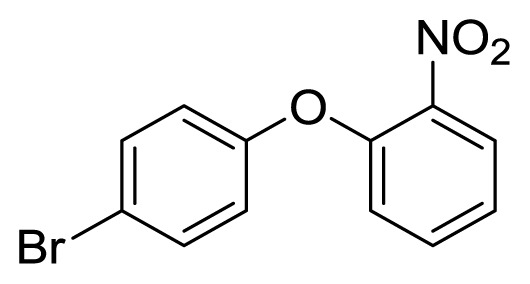 **3i**	89.5 c, 91.6 d
10	H	Me	H	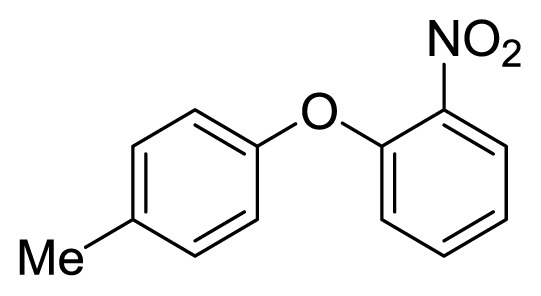 **3j**	96.2 c,d
11	H	OMe	H	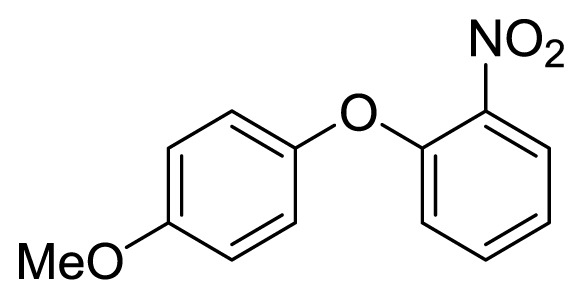 **3k**	99.0 c,d
12	H	H	H	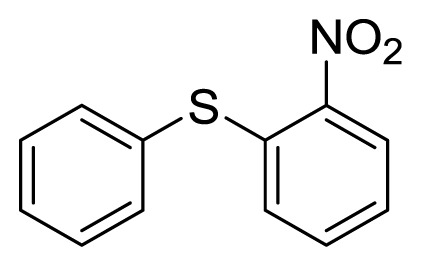 **3l**	94.4 c
13	H	Me	H	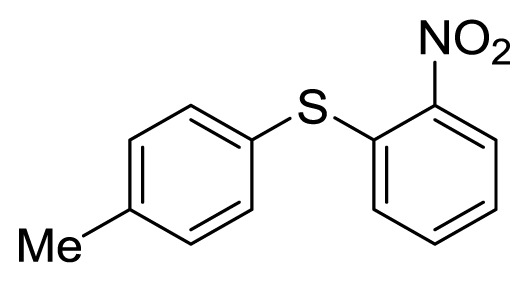 **3m**	97.7 c
14	H	Cl	H	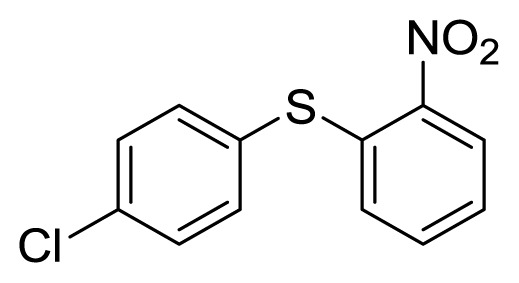 **3n**	89.4 d
15	Cl	Me	H	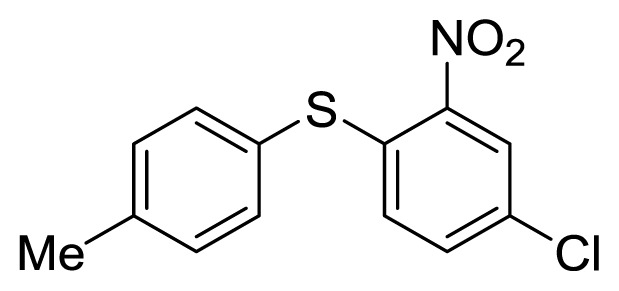 **3o**	94.7 c

a The reaction was performed at molar ratio of compound **1** and **2** at 1:1;

b isolated yield;

c 2-nitrochlorobenzene were used;

d 2-nitrofluorobenzene were used.

**Table 3:** Antifungal activities of **3a**–**o** to six phytopathogenic fungi.

	Antifungal activities (inhibition%)					
	
Compound	*Cytospora mandshurica*	*Curvularia lunata*	*Magnaporthe oryzae*	*Gloeosporium fructigenum*	*Alternaria lternata*	*Fusarium graminearum*
**3a**	41.96	6.65	2.10	11.94	0.00	0.00
**3b**	38.86	7.23	39.30	32.14	25.43	12.79
**3c**	18.56	47.60	38.64	24.77	30.52	25.46
**3d**	0.00	19.30	0.00	0.00	0.00	0.00
**3e**	9.85	0.00	1.37	0.00	0.00	0.00
**3f**	0.00	0.00	0.00	19.26	0.00	0.00
**3g**	16.07	40.37	37.24	25.70	55.95	28.11
**3h**	29.03	62.67	21.39	52.28	15.26	17.51
**3i**	31.08	37.37	14.48	14.69	30.52	35.03
**3j**	24.17	17.89	20.45	21.74	30.12	10.75
**3k**	21.26	14.46	24.82	24.77	27.06	0.00
**3l**	13.99	45.80	31.03	23.89	42.35	10.95
**3m**	48.18	22.30	44.85	33.96	0.12	34.31
**3n**	19.70	40.98	28.96	33.03	16.89	0.00
**3o**	58.76	44.46	48.75	39.73	28.71	42.31
Thiophannate-methyl	72.55	37.95	12.41	73.42	74.57	82.11
